# Aquatic Therapy as a Programmable Multisensory Environment for Arousal and Postural Control After Severe Acquired Brain Injury: A Perspective

**DOI:** 10.3390/brainsci16030344

**Published:** 2026-03-22

**Authors:** Andrea Calderone, Rosaria De Luca, Alessio Currò, Alessio Mirabile, Marco Piccione, Rocco Salvatore Calabrò

**Affiliations:** IRCCS Centro Neurolesi Bonino-Pulejo, S.S. 113 Via Palermo, C.da Casazza, 98124 Messina, Italy; rosaria.deluca@irccsme.it (R.D.L.); alessio.curro@irccsme.it (A.C.); alessio.mirabile@irccsme.it (A.M.); marco.piccione@irccsme.it (M.P.); roccos.calabro@irccsme.it (R.S.C.)

**Keywords:** severe acquired brain injury, disorders of consciousness, aquatic therapy, multisensory stimulation, arousal regulation, postural control

## Abstract

**Highlights:**

**What are the main findings?**

- Aquatic therapy is framed as a programmable multisensory rehabilitation medium in sABI.
- The Arousal–Alignment–Action loop offers testable links between state, posture, and action.

**What are the implications of the main findings?**

- Reporting core dosing parameters can improve transparency and study comparability.
- A minimal outcomes/confounders set enables pragmatic, cumulative evaluation of protocols.

**Abstract:**

**Background/Objectives**: Severe acquired brain injury (sABI) disrupts early rehabilitation because arousal fluctuates, trunk control is fragile, and agitation limits therapy tolerance; land-based practice is frequently constrained by fall risk and staffing. We aim to reframe aquatic therapy as a programmable multisensory environment to stabilize arousal and support axial alignment before conventional impairment targets are feasible. Here, programmable denotes the deliberate titration and reporting of water depth, turbulence or perturbation, temperature, body orientation, and flotation and manual support as intervention inputs. **Methods**: This perspective integrates principles from neurobehavioral assessment, motor control, and immersion physiology to propose the Arousal–Alignment–Action loop as a falsifiable model and to define manipulable aquatic inputs (water depth, turbulence or perturbation, temperature, body orientation, and flotation and manual support) as dosing parameters. We outline a pragmatic testing ladder (within-session micro-experiments, feasibility studies, and embedded evaluations) and a minimal outcomes and confounder set to support cumulative evidence. **Results**: The framework links state regulation to alignment and goal-directed behavior, specifies predictions that can fail, and highlights boundary conditions (sedation, autonomic instability, pain, recent surgery or wounds, and cervical or cardiopulmonary constraints). A minimal outcome package spanning arousal/responsiveness, trunk control, behavioral dysregulation, participation/tolerance, and basic physiology is proposed, with optional objective adjuncts for mechanism-oriented studies. **Conclusions**: Treating water as a measurable and titratable medium, rather than a generic modality, may reduce early intensity bottlenecks and improve implementability and comparability of aquatic neurorehabilitation research in medically stable sABI; however, the model is intended as hypothesis-generating until supported by stronger direct clinical evidence.

## 1. Introduction

Severe acquired brain injury (sABI), whether traumatic, vascular, or hypoxic, often leaves patients in prolonged disorders of consciousness (DoC) or with severely reduced goal-directed behavior. In the early phase, the main barrier to rehabilitation is frequently an unstable neurobehavioral state rather than the absence of impairment targets: arousal fluctuates, trunk control is fragile, and agitation can abruptly curtail therapy [1,2]. On land, these factors create an intensity bottleneck. Sitting and standing demand heavy guarding; sessions stop quickly when arousal drops or dysregulation escalates, and clinicians must trade practice dose against fall risk and staff burden. Sedatives and analgesics may improve safety but can blunt responsiveness, while poor axial alignment increases energy cost and can mask emerging purposeful actions.

Agitated behaviors are common during inpatient TBI rehabilitation, especially in post-traumatic amnesia, and guidance emphasizes structured assessment and non-pharmacological strategies despite heterogeneous evidence [3,4,5]. Multisensory stimulation approaches for DoC have also been explored, but reviews highlight variable protocols, modest methodological quality, and inconsistent outcomes that blend state, behavior, and function without a shared framework [6,7]. This aligns with broader efforts in coma therapeutics to connect mechanistic hypotheses, measurement, and trial design and to adopt common data elements so early-phase studies can be compared and confounders captured consistently [8,9].

Aquatic therapy is often framed as “hydrotherapy” for generic exercise. For sABI, that framing is incomplete. Much of the prior aquatic rehabilitation literature has emphasized mobility, balance, conditioning, and exercise capacity [10,11,12], whereas the clinical problem in early sABI is often one of unstable state regulation, limited postural organization, and poor tolerance for goal-directed engagement. Water can instead be treated as a programmable multisensory medium in which vestibular, proprioceptive, thermal, and interoceptive signals are titrated while the consequences of postural error are reduced. Here, programmable means that water depth, temperature, turbulence or perturbation, body orientation, flotation and manual support, task contingency, and session duration are deliberately set, recorded, and progressively adjusted as intervention inputs rather than delivered as nonspecific exposure. These programmable aquatic inputs therefore become manipulable levers to engineer graded exposure that targets arousal regulation, postural alignment, and task-oriented action before conventional strength or gait goals are realistic. The novelty of the present perspective is not to claim entirely new aquatic physiology, but to connect contemporary models of consciousness recovery with a parameterized view of the aquatic environment as a therapeutic medium, thereby linking defined inputs to measurable changes in arousal regulation, postural alignment, and task-oriented action, together with a minimal feasible outcome set [13]. At the current stage, this proposal should be read as hypothesis-generating and boundary-conditioned, given the still-limited direct evidence base in sABI.

In practical terms, this work follows a single trajectory: programmable aquatic inputs (e.g., water depth, temperature, turbulence or perturbation, body orientation, and flotation and manual support) are delivered through identifiable sensory and biomechanical channels; these channels are hypothesized to converge on an Arousal–Alignment–Action loop that shapes arousal regulation, postural alignment, and task-oriented action; and the framework is then translated into measurable behavioral, physiological, and clinical outputs. This overview is intended to orient the reader from mechanism, to dosing, to testing, and finally to implementation.

## 2. From Modality to Medium: Aquatic Neurorehabilitation as State Engineering

This section reframes aquatic therapy from a generic modality to a programmable therapeutic medium, showing why environmental engineering matters before conventional impairment-based goals can be pursued.

### 2.1. Enrichment as a Clinical Construct, Not a Metaphor

Enrichment is often invoked in neurorehabilitation, but it can be operationalized as structured modification of the setting that increases opportunities for physical, cognitive, and social activity beyond formal therapy [14]. For sABI, the practical criterion is whether the environment increases wakeful time, exploration, and goal-directed interaction without increasing distress or risk.

This position also distinguishes the framework from generic sensory stimulation. The therapeutic unit is not exposure alone, but exposure that is parameterized, posturally scaffolded, and linked to observable behavioral output, which makes it more amenable to replication, confounder control, and cumulative interpretation across studies [6,7,14].

To dose and study enrichment, parameters such as sensory intensity and salience, predictability, action contingency, and the density of safe postural opportunities should be documented. Built-environment work suggests that space and affordances shape activity patterns and staff workflows even when protocols are unchanged [15]. Water adds a medium in which these affordances can be tuned session-by-session.

### 2.2. Constraint Reshaping: Buoyancy and Safety as Participation Multipliers

Constraint reshaping is central: immersion changes the cost of movement and the consequences of error. Buoyancy unloads the body and supports upright positions, while viscosity and drag slow destabilizing motions, reducing the penalty of failed balance reactions and enabling safer motor experimentation [10].

Safety is a dose determinant. When staff and patient perceive safety, sessions can shift from defensive guarding to coached exploration and higher active practice. Scoping work across neurological disability highlights diverse water-based interventions but rarely specifies the environmental parameters that might drive state change [11]. In stroke, meta-analyses suggest aquatic therapy can improve balance, supporting plausibility for postural training in water [12]. For sABI, the key is to link these dose opportunities to measurable changes in arousal and alignment, not just exercise capacity.

### 2.3. Why Severe Acquired Brain Injury Is the Stress Test for Enrichment Models

sABI is a stress test for any enrichment model because arousal can fluctuate within minutes and overload can trigger agitation or withdrawal. A usable medium must simultaneously provide multisensory drive to stabilize attention and sufficient postural support to prevent threat. If state and posture can be shifted through controllable inputs, that would be informative even when cortical networks are severely disrupted.

At the neurophysiological level, this hypothesis is compatible with current models of consciousness recovery in which the ascending reticular/arousal system, thalamic relays, basal forebrain, and distributed cortical networks jointly constrain wakefulness and responsiveness. Structural disruption of ascending arousal pathways has been demonstrated in acute traumatic disorders of consciousness, and longitudinal increases in brainstem–thalamic connectivity accompany recovery after traumatic coma [16,17,18]. In parallel, vestibular and somatosensory graviception jointly calibrate the internal estimate of body verticality, supporting the rationale for using buoyancy, depth, and therapist-guided body orientation to shape alignment rather than merely unload weight [19]. Resting-state imaging studies further suggest that impaired interactions between brainstem arousal systems and cortical awareness networks are biologically relevant in DoC [20].

Mechanistic plausibility also involves interoception. Immersion increases hydrostatic pressure and changes respiratory and cardiovascular loading, shifting autonomic balance and perceived body state. Water-related stimuli can increase cardiac vagal activity in time-locked ways [16]. In rehabilitation, this argues for treating thermal comfort, depth, and brief facial immersion (when safe) as adjustable inputs to tune arousal while therapists shape postural alignment and action initiation [21].

## 3. Four Sensory Channels, One Neurobehavioral Axis

### 3.1. Vestibular Loading Without Catastrophic Falls

This section breaks the aquatic environment into its main sensory and biomechanical channels, because similar behavioral outputs may emerge through partially distinct vestibular, proprioceptive, thermal, and interoceptive routes. Buoyancy permits vestibular dosing with lower early risk than land because head and trunk displacement carries less penalty. Depth, flotation and manual support, and task choice let clinicians titrate rotation amplitude and velocity while keeping the center of mass controllable; turbulence or therapist-generated perturbation can add graded challenges while water slows motion [22]. Measurable signature: time-locked change in Coma Recovery Scale-Revised (CRS-R) arousal items during a standardized sequence of supported head turns at chest depth [23,24]. Key confounder: vestibular agnosia or central vestibular deficits may blunt downstream behavioral effects [25].

### 3.2. Proprioceptive Reweighting Under Buoyancy

Buoyancy reduces axial load but preserves joint motion, shifting proprioceptive weighting and potentially unmasking trunk strategies constrained by fear, spasticity, or limited support. Hydrostatic pressure adds broad cutaneous input that may sharpen midline perception. Clinicians can grade this channel by changing depth, adjusting flotation and manual support during reaching tasks, and tapering assistance as postural alignment improves; turbulence or perturbation adds sensory noise that challenges rigid co-contraction. Measurable signature: reduced sway during a standardized sitting test as external support is decreased across sessions [26]. Key confounder: impaired sensory reweighting may predict non-response despite similar dose [27].

### 3.3. Thermal Modulation as Arousal Shaping

Water temperature shapes autonomic tone and the arousal window available for learning. Thermoneutral or mildly warm immersion may promote parasympathetic predominance and reduce guarding, whereas cooler water may increase alertness but also stress and shorten tolerance. Temperature, exposure time, and immersed surface can be titrated with gradual transitions. Measurable signature: increased heart rate variability (HRV), vagal indices and stronger respiration–cardiac coupling during thermoneutral head-out immersion [28]. Key confounder: individual thermoregulation can reverse HRV direction and mislead interpretation [29].

### 3.4. Hydrostatic Pressure and Interoception: The Neglected Pathway

Hydrostatic pressure continuously reshapes cardiopulmonary mechanics and interoceptive signaling: central blood shift, altered baroreflex loading, and increased work of breathing can stabilize or destabilize arousal depending on reserve. Interoceptive inference has been linked to conscious state regulation [30]. Pressure can be graded by depth and posture, paired with breath pacing, and (when clinically appropriate) brief facial immersion to recruit vagal reflexes. Measurable signature: increased vagally mediated heart rate variability (vmHRV) during chest-level immersion with a brief facial-immersion challenge [31]. Key confounder: paroxysmal sympathetic hyperactivity (PSH) or vasoactive drugs can dominate autonomic signals and require concurrent monitoring [32].

## 4. The Arousal–Alignment–Action Loop: Predictions That Can Fail

This section converts the proposed channels into an operational model, specifying how arousal regulation, postural alignment, and task-oriented action can be measured together and what findings would count against the framework. Importantly, the loop is intended to be operational rather than metaphorical. Arousal can be indexed behaviorally with repeated CRS-R/SECONDs sampling and, in mechanistic substudies, with EEG command-following paradigms or somatosensory evoked potentials; alignment can be indexed with supported sitting time, trunk angle, sway, and standardized trunk scales; and action can be indexed with command-following accuracy, purposeful reaching, object use, and session engagement. When available, resting-state or task-based neuroimaging and portable hemodynamic measures can provide convergent biological signals for mechanism-oriented studies [20,21,22,23].

### 4.1. Model Definition

**Figure 1:**
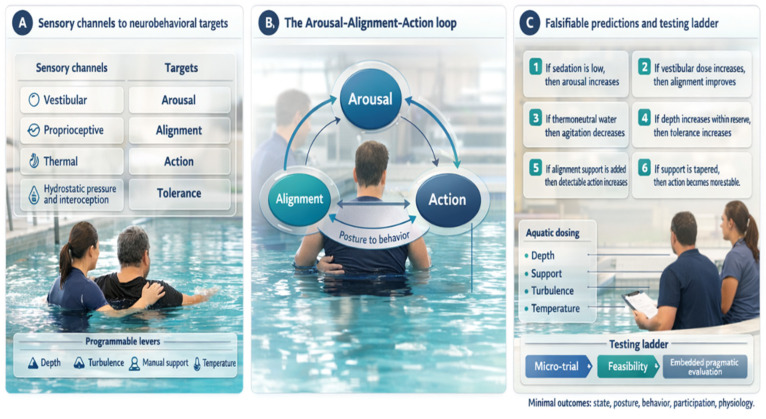
Programmable multisensory medium and Arousal–Alignment–Action model in severe acquired brain injury.

**Figure 2:**
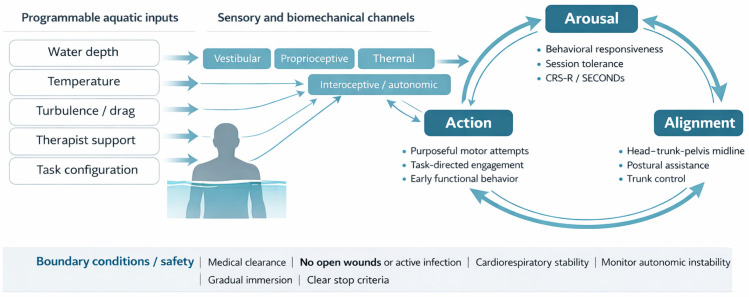
Programmable aquatic inputs, sensory and biomechanical channels, and the Arousal–Alignment–Action loop in sABI. The schematic reads from left to right: programmable aquatic inputs are delivered through sensory and biomechanical channels and are hypothesized to influence arousal regulation, postural alignment, and task-oriented action, thereby shaping clinically observable outputs.

Arousal is the capacity to sustain wakefulness and produce reliable responses to standardized prompts; in sABI it must be sampled repeatedly because fluctuations and motor limits can mimic absent awareness [33]. State can be tracked with repeated CRS-R totals and subscores, interpreting change against minimal clinically important difference estimates [34]. Alignment refers to head–trunk–pelvis organization relative to gravity enabling gaze, ventilation, and limb use; it can be quantified by supported sitting time, trunk angle, and sway, noting that CRS-R performance differs between upright and supine positions [35]. Action is any goal-directed attempt linking intention to movement, sampled as purposeful response counts and, when time is limited, with brief tools such as the Simplified Evaluation of Disorders of Consciousness (SECONDs) [36]. The coupled loop and programmable levers are summarized in Figure 1, while Figure 2 makes explicit the direction from programmable aquatic inputs, through sensory and biomechanical channels, to the Arousal–Alignment–Action loop and its observable behavioral and clinical outputs. Figure 1 and Figure 2 were prepared with the assistance of ChatGPT (GPT-4 multimodal version, including image-generation functionality) solely for conceptual illustration; the generated output was critically reviewed and edited by the authors before inclusion in the manuscript.

To make the term programmable clinically usable, the principal aquatic inputs should be described as intervention variables rather than impressionistic descriptors. Table 1 organizes the main inputs according to how they are dosed, the hypothesized sensory and biomechanical target within the Arousal–Alignment–Action loop, candidate readouts, and immediate safety flags.

### 4.2. Coupling Rules

Within the loop, state shapes posture through tone and multisensory integration. Depth and support can raise arousal by reducing threat and providing rhythmic vestibular and tactile input, enabling a more neutral trunk. Alignment then constrains what can be perceived and expressed: midline head control improves visual targeting and reduces competing reflex patterns, making responses easier to detect. Successful actions provide salient feedback and can stabilize attention, while immersion-related autonomic shifts can be sampled with HRV during therapy [37].

### 4.3. Six Falsifiable Predictions

Six falsifiable predictions follow. Prediction 1: With lighter sedation, weekly CRS-R gains will exceed time-matched land sessions; this advantage will attenuate when sedative dose remains high. Prediction 2: Stepwise increases in vestibular and turbulence dose will improve supported sitting time and trunk midline, unless cervical instability or severe vestibular hypofunction is present. Prediction 3: Thermoneutral immersion will reduce agitation episodes and early termination, unless pain or infection is driving dysregulation. Prediction 4: Within physiological reserve, greater depth (hydrostatic pressure) will increase tolerance (active minutes), but not in dysautonomia, fluid overload, or ventilatory dependence. Prediction 5: Adding external alignment support will acutely increase SECONDs and CRS-R motor performance, but effects will vanish when support is removed (performance vs. learning). Prediction 6: Choice-based reaching paired with tapered support will reduce sway variability and increase purposeful actions when aphasia, apraxia, and neglect are accommodated.

### 4.4. A Pragmatic Testing Ladder

Testing can build from feasibility evidence: aquatic therapy has been delivered during post-acute severe traumatic brain injury in a randomized trial, supporting practicality and acceptability [38]. Next, within-individual micro-trials can manipulate one channel at a time (e.g., depth, flotation and manual support, or temperature) with rapid alternation and repeated pre-post state and alignment measures to estimate immediate signal and variability [39]. Small-cohort feasibility studies should then focus on recruitment, adverse events, fidelity, and the stability of outcome collection under routine staffing, using guidance for pilot and pragmatic designs [40,41]. Finally, embedded pragmatic evaluations can compare state-engineering protocols with usual care within services, prioritizing transportability and minimal burden, consistent with pragmatic trial frameworks in neurology [42]. Where resources allow, these micro-trials can pair behavioral outcomes with concurrent or same-day objective measures (e.g., EEG reactivity/command-following, SEP amplitudes, or imaging-derived network markers) to test whether behavioral change is accompanied by measurable neural signal change [20,21,22,23].

## 5. Discussion: Boundaries, Confounders, and a Minimal Outcomes Package

The discussion narrows the framework to the conditions under which it can be used responsibly, the confounders that can distort interpretation, and the minimum measurement set needed for clinically meaningful accumulation of evidence.

### 5.1. Boundary Conditions

Boundary conditions define who should not receive aquatic state-engineering and when sessions should be deferred. Contraindications include uncontrolled seizures, non-healed wounds, active skin infection, and uncontrolled incontinence in shared pools [43]. Severe impulsive aggression is a deferral point when safety cues cannot be followed. Structured screening and an explicit stop plan help standardize decisions and avoid ad hoc exclusions [44]. Because immersion increases venous return and pulmonary blood volume, decompensated heart failure, unstable arrhythmia, and high oxygen demand should preclude immersion until stability improves [45]. This scope should be stated explicitly: the present framework is intended for medically stable, screened patients and should not be read as support for routine pool exposure in the acute ICU phase. Recent neurosurgical procedures, external drains, non-healed lines or stomas, unstable fractures, or unresolved infection increase both infection-control burden and transfer risk and should defer immersion until the treating team confirms stability [43,44,46].

Session rules should specify physiological thresholds and a rapid extraction plan. New desaturation, sustained tachycardia, marked blood pressure lability, or escalating autonomic storms should prompt pausing and, if persistent, stopping. Tracheostomy or ventilation requires trained staff, secure fixation, waterproofing, and immediate suction access, limiting early candidacy [46]. Shunt-treated hydrocephalus is not an automatic exclusion, but recent revision or skin compromise should delay immersion [47]. Paroxysmal sympathetic hyperactivity (PSH) warrants conservative dosing of depth and temperature and postponement during clustered episodes or medication transitions [32]. Autonomic instability deserves explicit monitoring because head-out immersion modifies venous return, pulmonary blood volume, and baroreflex loading. In dysautonomia or PSH, entry should be gradual, depth limited, thermoneutral water preferred, and cardiorespiratory monitoring intensified whenever clinically indicated [32,45].

### 5.2. Confounders Checklist

Interpretation is threatened by expected confounding early in neurorehabilitation. Sedation and analgesia changes can shift apparent arousal and agitation independent of aquatic exposure [48]. Spontaneous recovery, time since injury, and co-interventions (verticalization, orthoses, stimulants) shift baseline. Sleep disruption, pain, infection, constipation, and urinary retention can mimic dysregulation or low engagement, and assessment position matters because scores can differ between supine testing and supported sitting.

A brief pre-session checklist can standardize capture of positioning, recent medication changes, sleep opportunity, pain behaviors, infections, and medical instability before behavioral testing [49]. The same form can log exposure parameters (water depth, temperature, turbulence or perturbation, body orientation, flotation and manual support), active minutes, interruptions, and staff ratio; recording therapist identity supports pragmatic analyses and safer handovers.

### 5.3. Minimum Outcome Battery

A minimal outcomes battery should map onto the Arousal–Alignment–Action framework while remaining feasible (Table 2). State can be measured with CRS-R or SECONDs at baseline and weekly, with pre-post sampling in a subset to capture within-session shifts [50]. Postural alignment can be tracked with supported sitting tolerance and a validated trunk scale (e.g., Trunk Recovery Scale) assessed weekly in a standardized body orientation [51]. Behavioral dysregulation can be tracked with the Agitated Behavior Scale alongside pain and medication notes [52]. Participation and tolerance can be captured as completed sessions, active minutes, and premature termination; optional physiology includes routine heart rate, blood pressure and HRV in a subset [53]. Objective adjuncts are not required for routine implementation, but in nested mechanistic studies EEG command-following/reactivity, SEP amplitudes, and neuroimaging or portable hemodynamic measures can strengthen inference by testing whether behavioral gains co-occur with measurable neural signal change [20,21,22,23].

### 5.4. Clinical Relevance and Implementation Value

In practice, aquatic therapy in sABI is often used pragmatically or opportunity-driven, with the pool treated as a generic adjunct rather than as a parameterized intervention. An explicit framework matters because it makes visible what is being manipulated (e.g., water depth, turbulence or perturbation, body orientation, flotation and manual support, and work-rest structure), why that choice is being made, which sensory and biomechanical channel is being targeted, and how exposure is being increased or reduced over time. This improves clinical reasoning, documentation, interdisciplinary communication, and safety planning, while also making protocols more reproducible and more comparable across services and studies [11,43,44]. Accordingly, the framework is already clinically usable as an organizing scaffold for describing, dosing, and reporting aquatic sessions in appropriately screened patients, even though its more specific mechanistic predictions remain hypothesis-generating until tested directly.

### 5.5. Limitations

This framework should be read against important limitations. Direct randomized evidence in sABI remains sparse, and the single available post-acute trial does not by itself establish efficacy across the heterogeneous sABI population [38]. Implementation is also constrained by practical barriers, including pool access, staff expertise, transfer logistics, medical screening, and the monitoring capacity required for patients with tracheostomy, autonomic instability, or high medical complexity [43,45,46]. Finally, several proposed pathways, especially those linking immersion-related interoceptive or autonomic effects to changes in conscious state, are supported more by physiological plausibility and indirect evidence than by direct causal neural demonstration in this specific population [16,17,18,20,30,37]. Recognizing these limits does not weaken the framework; it clarifies that its immediate role is to organize clinical reasoning and structured testing rather than to claim a definitive mechanism or superiority.

## 6. Conclusions

In closing, the framework is intended less as a claim about a unique modality and more as a way to make early aquatic rehabilitation conceptually explicit, clinically reportable, and empirically testable. This perspective treats aquatic therapy as a programmable multisensory medium for early sABI rehabilitation, where environmental levers can be dosed to stabilize arousal regulation, support postural alignment, and elicit task-oriented action while reducing the penalty of postural error. The Arousal–Alignment–Action loop frames these variables as coupled, measurable, and falsifiable rather than anecdotal. With explicit boundary conditions, minimal confounder capture, and a feasible outcome battery, routine sessions can already be described and reported with greater precision, allowing aquatic therapy to function clinically as a structured organizing scaffold in appropriately screened patients. Micro-trials can quantify the immediate signal, followed by feasibility studies and pragmatic evaluations embedded in standard pathways. However, the mechanistic linkages and predictive claims proposed here remain hypothesis-generating until supported by stronger direct empirical evidence in sABI. If workable in this population, the same parameterized logic may also inform other controlled rehabilitation environments designed to engineer participation when conventional therapy cannot yet scale.

## Figures and Tables

**Table 1:** Programmable aquatic parameters, hypothesized targets, candidate readouts, and safety considerations within the Arousal–Alignment–Action framework.

Programmable Input	How to Dose/Report	Hypothesized Primary Target	Candidate Readouts	Main Safety Flags
Water depth	Anatomical landmark and approximate immersed proportion	Buoyancy and hydrostatic pressure; cardiopulmonary/interoceptive loading	CRS-R/SECONDs pre-post; active minutes; HR/BP/SpO_2_; supported sitting time	Cardiopulmonary reserve, PSH/dysautonomia, oxygen demand
Water temperature	Exact range, acclimation period, and exposure duration	Autonomic tone, thermal comfort, guarding/spasticity modulation	Agitation/tolerance, HRV, premature termination, engagement	Impaired thermoregulation, fever/infection, autonomic lability
Turbulence or perturbation	Source, amplitude, direction, frequency, and progression	Vestibular challenge, sensory reweighting, and postural alignment	Sway, trunk correction frequency, protective responses, purposeful action	Cervical instability, vestibular intolerance, impulsivity
Flotation and manual support	Body segment supported, assistance level, flotation device use	Threat reduction, postural alignment, and error control	Support level achieved, motor items on CRS-R, reaching quality	Over-assistance masking true performance
Body orientation	Supine, semi-seated, sitting, standing, or turning sequence	Vestibular load, visual access, respiratory mechanics, and postural alignment	Upright tolerance, trunk angle, command-following, gaze stabilization	Orthostatic intolerance, airway/tracheostomy management
Task contingency	Cue type, choice structure, feedback, and object interaction	Salience, attention, and goal-directed behavior	Purposeful response count, command accuracy, object use, engagement	Aphasia, apraxia, neglect, sensory overload
Session duration and work-rest structure	Active minutes, breaks, total session time, and interruption criteria	Fatigue accumulation, autonomic burden, learning window	Completion rate, breaks, HR/BP recovery, therapist-rated tolerance	Pain, fatigue, desaturation, delayed dysregulation
Programmable input	How to dose/report	Hypothesized primary target	Candidate readouts	Main safety flags
Water depth	Anatomical landmark and approximate immersed proportion	Buoyancy and hydrostatic pressure; cardiopulmonary/interoceptive loading	CRS-R/SECONDs pre-post; active minutes; HR/BP/SpO_2_; supported sitting time	Cardiopulmonary reserve, PSH/dysautonomia, oxygen demand
Water temperature	Exact range, acclimation period, and exposure duration	Autonomic tone, thermal comfort, guarding/spasticity modulation	Agitation/tolerance, HRV, premature termination, engagement	Impaired thermoregulation, fever/infection, autonomic lability
Turbulence or perturbation	Source, amplitude, direction, frequency, and progression	Vestibular challenge, sensory reweighting, and postural alignment	Sway, trunk correction frequency, protective responses, purposeful action	Cervical instability, vestibular intolerance, impulsivity
Flotation and manual support	Body segment supported, assistance level, flotation device use	Threat reduction, postural alignment, and error control	Support level achieved, motor items on CRS-R, reaching quality	Over-assistance masking true performance
Body orientation	Supine, semi-seated, sitting, standing, or turning sequence	Vestibular load, visual access, respiratory mechanics, and postural alignment	Upright tolerance, trunk angle, command-following, gaze stabilization	Orthostatic intolerance, airway/tracheostomy management
Task contingency	Cue type, choice structure, feedback, and object interaction	Salience, attention, and goal-directed behavior	Purposeful response count, command accuracy, object use, engagement	Aphasia, apraxia, neglect, sensory overload
Session duration and work-rest structure	Active minutes, breaks, total session time, and interruption criteria	Fatigue accumulation, autonomic burden, learning window	Completion rate, breaks, HR/BP recovery, therapist-rated tolerance	Pain, fatigue, desaturation, delayed dysregulation

Notes: Inputs should be reported at the session level to permit dose–response interpretation. Anatomical landmarks for depth, exact temperature range, assistance level, and interruption criteria should be prespecified whenever possible. Abbreviations: AAA, Arousal–Alignment–Action; CRS-R, Coma Recovery Scale-Revised; HRV, heart rate variability; PSH, paroxysmal sympathetic hyperactivity; SpO_2_, peripheral oxygen saturation.

**Table 2:** Minimum outcome battery and minimal confounder capture for aquatic state engineering protocols in severe acquired brain injury.

Domain	Core Measures (Minimum Set)	Optional Add-Ons (Mechanistic/Refinement)	Recommended Timing	Minimal Confounders to Document	Practical Notes
State (arousal/responsiveness)	Coma Recovery Scale-Revised (CRS-R): total and key subscores, with fixed assessor and standardized cues	Simplified Evaluation of Disorders of Consciousness (SECONDs) for rapid sampling; brief pupillary reactivity note if available	Baseline (within 7 days pre-start) Weekly (same weekday) Pre-post for a subset of sessions	Sedation and analgesia changes within 24 h; stimulant use; sleep opportunity; assessment body position	Use the same testing position and environmental conditions when possible; record position if it changes
Posture Alignment	Supported sitting tolerance (minutes) with standardized support level; Trunk Recovery Scale (TRS) or equivalent validated trunk scale	Instrumented sway during unstable sitting when feasible; simple trunk angle estimate (video or wearable) for midline tracking	Baseline Weekly Pre-post for a subset when dosing is changed	Pain behaviors; spasticity management changes; orthoses or seating supports; therapist assistance level	Anchor support level to an ordinal scale so dose and assistance are comparable between therapists
Behavior (dysregulation/agitation)	Agitated Behavior Scale (ABS): peak score and mean score over 24 h when feasible; session termination reason	Richmond Agitation-Sedation Scale (RASS) as context when used clinically; brief delirium screen where service policy mandates	Baseline Weekly Daily tracking where routine nursing scores exist	Infection markers and fever; constipation or urinary retention; medication changes; environmental triggers	Interpret change alongside clinical precipitants; avoid attributing reductions to the medium during acute medical deterioration
Participation (tolerance/dose)	Active minutes per session; session completion; number and duration of unplanned breaks	Therapist perceived exertion of the patient or staff assistance intensity; task-specific repetitions when measurable	Every session Weekly summary	Staffing ratio; therapist identity; transport and scheduling disruptions; co-interventions on the same day	Define active minutes consistently (time engaged in task, excluding setup and passive rest) and document interruptions
Physiology (autonomic feasibility)	Heart rate and blood pressure pre, during, and post session; oxygen saturation where routine	Heart rate variability (HRV) 5 min segments at rest and during immersion; respiratory rate; skin temperature	Every session for routine vitals Pre-post for HRV subset	Paroxysmal sympathetic hyperactivity episodes; vasoactive drugs; dehydration or fluid overload risk; ambient and water temperature	Use consistent sensor placement and segment selection; flag artifacts and arrhythmia that invalidate HRV interpretation
Exposure fidelity (intervention dose)	Depth; temperature; turbulence/perturbation; body orientation; flotation/manual support; task category; session duration.	Video-assisted coding of support and perturbations; wearable motion sensors for movement counts when relevant	Every session	Pool context changes (crowding, noise); therapist learning effects; protocol deviations	Treat these parameters as the manipulable inputs for the model; without them, interpretation of outcomes is weak
Global confounder set (required for interpretation)	Time since injury and rehabilitation phase; key comorbidities affecting arousal or posture; major co-interventions (verticalization, pharmacologic stimulants)	None	Baseline Weekly update Event-based updates when medication or medical status changes	Spontaneous recovery trajectory; sleep–wake disruption; pain and infection; assessment position effects	Keep this set short and consistently captured to enable pragmatic analyses and between-site comparability

Notes: Core measures represent the minimum set recommended for comparability. Optional add-ons can be used in a subset to probe mechanisms or improve precision; objective neurophysiological or imaging adjuncts are optional and intended for mechanistic subsets rather than routine use. Consistent assessment position and documentation of recent medication or medical changes are critical for interpretation. Abbreviations: ABS, Agitated Behavior Scale; CRS-R, Coma Recovery Scale-Revised; HRV, heart rate variability; RASS, Richmond Agitation-Sedation Scale; sABI, severe acquired brain injury; SECONDs, Simplified Evaluation of Disorders of Consciousness; TRS, Trunk Recovery Scale.

## Data Availability

No new data were created or analyzed in this study.
